# The Evaluation of Zoomed Echo-Planar Diffusion-Weighted Magnetic Resonance Imaging With Two-dimensional Spatial-Selective Radiofrequency Excitation Pulses in Patients With Hilar Cholangiocarcinoma

**DOI:** 10.3389/fonc.2022.816008

**Published:** 2022-06-22

**Authors:** Jingjing Liu, Mengyue Huang, Yanan Ren, Man Xu, Yinhua Li, Jingliang Cheng, Jinxia Zhu

**Affiliations:** ^1^ Department of Magnetic Resonance Imaging, The First Affiliated Hospital of Zhengzhou University, Zhengzhou, China; ^2^ MR Collaboration, Siemens Healthcare Ltd., Beijing, China

**Keywords:** hilar cholangiocarcinoma, zoomed EPI, diffusion-weighted imaging, magnetic resonance imaging, image quality

## Abstract

**Background:**

We aimed to investigate the feasibility and application of using the zoomed diffusion-weighted echo-planar imaging (z-EPI DWI) sequences for hilar cholangiocarcinoma assessment compared with conventional single-shot EPI diffusion-weighted imaging (c-EPI DWI).

**Methods:**

Both c-EPI DWI and z-EPI DWI were preoperatively performed in 16 patients with histopathologically-confirmed hilar cholangiocarcinoma. A two-dimensional spatial-selective radiofrequency (RF) pulse was applied to the z-EPI DWI using an echo-planar transmit trajectory. Anatomic structural visualization, lesion conspicuity, artifact presence and overall image quality were evaluated and compared between the two sequence images. The ratio of differences regarding hilar cholangiocarcinoma lesion sizes measured on T2-weighted imaging (T_2_WI) and diffusion-weighted imaging (DWI) were compared from both EPI techniques. The DW images for tumor involvement of the bile duct were reviewed based on histopathological examination of the surgical intraoperative evaluation. ADC measurements of DWIs in the hilar cholangiocarcinoma lesions were conducted.

**Results:**

The hepatic hilar region was better delineated by visualization of anatomical structures, lesion conspicuity and overall image quality using the z-EPI DWI and these analyses were compared with the c-EPI DWI method (all p<0.05). Better lesion delineation of bile duct walls and lumens was noted in four patients with z-EPI DWI compared with those of c-EPI DWI. No significant differences were noted between the two image datasets for artifacts (p=0.876). The ratio of differences regarding hilar cholangiocarcinoma lesion sizes was significantly lower (p= 0.018) on T_2_WI and DWI, as determined by the z-EPI DWI than that determined by the c-EPI method. The use of z-EPI DWI resulted in the accurate diagnosis of the Bismuth-Corlette classification of 15 tumors (15/16, 93.75%), whereas the use of c-EPI DWI resulted in correct diagnosis of 12 tumors (12/16, 75.00%). There were no significant differences between c-EPI DWI and z-EPI DWI in the ADC values of hilar cholangiocarcinoma lesions (p= 0.48).

**Conclusion:**

z-EPI DWI resulted in remarkable image quality improvements for hilar cholangiocarcinoma. The ability to detect and delineate lesions using z-EPI DWI was superior to that of c-EPI DWI.

## Background

Diffusion-weighted imaging (DWI) has been implemented to detect and characterize various abdominal lesions and offers high contrast between tumors and surrounding normal tissues (1). In recent years, DWI has been used to assess bile duct obstructions in several hilar cholangiocarcinoma studies (2–4). DWI can improve the sensitivity of evaluating the extent of tumor spread as well as the degree of bile duct and liver invasion (3). However, DW images obtained with conventional single-shot echo-planar imaging (ss-EPI) are often degraded by poor spatial resolution, susceptibility artifacts and image blurring (5–7).

Two-dimensional spatially selective radiofrequency excitation pulses combined with reduced field of view (FOV) ('zoomed') imaging along phase-encoding directions can lead to superior image quality with reduced spatial distortions and artifacts and improved identification of small anatomic structures compared with that of conventional EPI (c-EPI) for DWI (8, 9). This novel sequence focuses on a specific organ instead of unnecessary imaging of entire upper abdominal regions. Recent studies using this method mainly concentrate on the pancreas, kidney, prostate, spinal cord and head and neck regions (8–16). However, there have been no studied on the application of zoomed EPI (z-EPI) DWI to examine the hepatic hilar region. Therefore, in the present study, the potential advantages of using z-EPI DWI of hilar cholangiocarcinoma examinations were investigated and compared with those of using c-EPI DWI.

## Materials and Methods

### Study Population

This retrospective study was approved by the Institutional Review Board of the First Affiliated Hospital of Zhengzhou University with waiver of patient informed consent. The information was derived from the surgical database of the First Affiliated Hospital of Zhengzhou University between August 2018 and December 2019 using “hilar cholangiocarcinoma” as the search phrase. The inclusion criteria were as follows: (a) preoperative magnetic resonance imaging (MRI) examinations of the upper abdomen for biliary evaluations, including the use of c-EPI DWI and z-EPI DWI; (b) surgery at our institution within 2 weeks following MRI; (c) histologically confirmed hilar cholangiocarcinoma and (d) no prior oncological treatment, such as chemotherapy or radiotherapy. Finally, 16 patients (mean age 58 ± 12 years, range 25-73, 12 men, and four women) were included in the study population.

### MRI Technique

The abdominal MRI study was performed on 16 patients using a 3T whole-body MR system (MAGNETOM Prisma, Siemens Healthcare, Erlangen, Germany) with an 18-channel phased-array body coil as the receiver coil. Each patient underwent both c-EPI DWI and z-EPI DWI (b values=50 and 800 s/mm2) of the hepatic hilus. The conventional sequences included a coronal breath-hold T2-weighted half-Fourier single-shot turbo spin echo sequence (HASTE), an axial fat-suppressed respiratory-triggered (RT) T2-weighted turbo spin echo sequence (TSE), and a three-dimensional volumetric interpolated breath-hold examination (VIBE) sequence was repeated four times for the T1-weighted dynamic contrast-enhanced (DCE) imaging (pre-enhanced phase, arterial phase, portal vein phase, and delay phase). After pre-enhanced phases, 0.1 mmol/kg of Gd-DTPA was injected at a rate of 2 mL/s. The detailed imaging parameters are listed in **Table 1**. Nine patients underwent DCE MRI.

**Table 1:** Magnetic resonance imaging parameters.

Imaging parameters	c-EPI DWI	z-EPI DWI	Coronal T_2_WI	Axial T_2_WI	T1WI/DCE
TR [ms]	4500	2000	1400	3100	3.9
TE [ms]	56	61	67	87	1.89
FOV [mm^2^]	350 × 292	230 × 120	360 × 360	380 × 380	380 × 309
Reconstructed voxel size (mm^3^)	2.2 × 2.2 × 5	1.5 × 1.5 × 5	1.4 × 1.4 × 5	1.2 × 1.2×5	0.7 × 0.7 × 3
Acquisition matrix	158 × 121	154 × 50	256 ×256	320 × 320	288 × 187
Slice thickness [mm]	5	5	5	5	3
b-values [s/mm^2^]	50/800	50/800	/	/	/
Parallel acceleration factor	2	2	3	2	2
Bandwidth (Hz/px)	1978	1248	698	710	1090
Respiration control	Free-breathing	Trigger	Breath-hold	Trigger	Breath-hold
Acquisition time	1min52s	3min18s~6min11s (depending on breathing pattern)	28s	3min44s~4min28s (depending on breathing pattern)	17s
					

c-EPI, conventional echo-planar imaging; z-EPI, zoomed echo-planar imaging; DWI, diffusion-weighted imaging; T_2_WI, T2-weighted imaging; T_1_WI, T1-weighted imaging; DCE, dynamic contrast-enhanced.

### Qualitative Image Analysis

All DW images were evaluated independently on a PACS workstation by two experienced radiologists (with 5 and 8 years of experience in abdominal MRI, respectively) in a randomized fashion. Each reader independently ranked the images acquired using both EPI sequences with regard to image quality and overall scan preference. Both EPI sequences were evaluated with a 4 point scale for 1) anatomical structure visualization (1, poorly visualized anatomy and non-diagnostic; 2, fairly delineated hepatic hilus zone with margin blurring; 3, optimal delineation of hepatic hilus zones with sharp margins; 4, excellent sharpness of hepatic hilus zone), 2) lesion conspicuity (1, lesion not detectable; 2, simply lesion-to-background contrast recognized; 3, intermediate lesion-to-background contrast or high contrast with indistinct lesion margins seen; 4, excellent lesion-to background contrast and clear lesion margins), 3) artifacts (1, severe and non-diagnostic; 2, moderate; 3, mild; 4, absent) and 4) overall image quality as a sum of the aforementioned 3 parameters. The readers initially evaluated only the c-EPI DW images and subsequently the z-EPI DWI using the same criteria. For each DWI, only high b-value images (b=800 s/mm^2^) were analyzed.

### Quantitative Analysis

The hilar cholangiocarcinoma lesion sizes were measured on T_2_WI and DWI acquired from both EPI sequences. First of all, the maximum diameter of the lesion on T_2_WI was measured. Then, the lesion diameter was measured according to hyperintense signal on z-EPI DWI and c-EPI DWI, respectively. Further, the ratio of differences regarding lesion sizes was calculated according to the following formula:(Size_T2WI_-Size_DWI_)/Size_T2WI._

The measurements of ADC values of hilar cholangiocarcinoma lesions were independently performed by a single different radiologist with 6 years of experience in radiology. The ADC values of the lesions were obtained by manually placing a circular region of interest (ROI) on the ADC maps acquired from both the c-EPI and z-EPI DWI sequences. ROIs were placed at near-identical locations on both sequences with care to avoid vessels and bile ducts. ROIs in the lesions were made 3 times. The measurements of ADC values were performed in 8 patients because of it was difficult to accurately measure some lesions with relatively small sizes.

### Image Analysis

Two different abdominal radiologists with 6 and 12 years of experience in liver MR imaging study interpretation, respectively, independently reviewed DW images for tumor involvement in the bile duct. They were blinded to the surgical and pathological findings but were informed that the study population had hilar cholangiocarcinoma. The readers reviewed DW images combined with conventional T_2_WI, and determined the tumor biliary extension according to the Bismuth-Corlette classification in stages I, II, IIIa, IIIb and IV (17). The final diagnosis of all bile duct tumors was based on histopathological examination following intraoperative evaluation of the extent of bile duct tumor infiltration.

### Statistical Analysis

Anatomical and structural visualization, lesion conspicuity, the presence of artifacts and overall image quality scores of the images acquired from both EPI sequences were compared using the Wilcoxon signed-rank test. The comparisons were made using the average scores between the two readers. An inter-reader agreement for each assessed qualitative evaluation was used for weighted κ statistics. An inter-reader agreement was considered slight for κ=0.00-0.20, fair for κ=0.21-0.40, moderate for κ=0.41-0.60, substantial for κ=0.61-0.80 and almost perfect for κ=0.81-1.00. The means for lesion sizes from both EPI sequences and T_2_WI were compared using the one-way ANOVA test. Statistical differences between the ratio of differences and ADC values were evaluated using the Student’s *t* test. All statistical analyses were performed using SPSS statistics 19 (IBM, Armonk/NY, USA). A *P*-value <0.05 was considered for significant differences.

## Results

All the hepatic hilar regional MR examinations, including z-EPI DWI and c-EPI DWI were successfully performed.

### Qualitative Image Analysis

**Figure 1:**
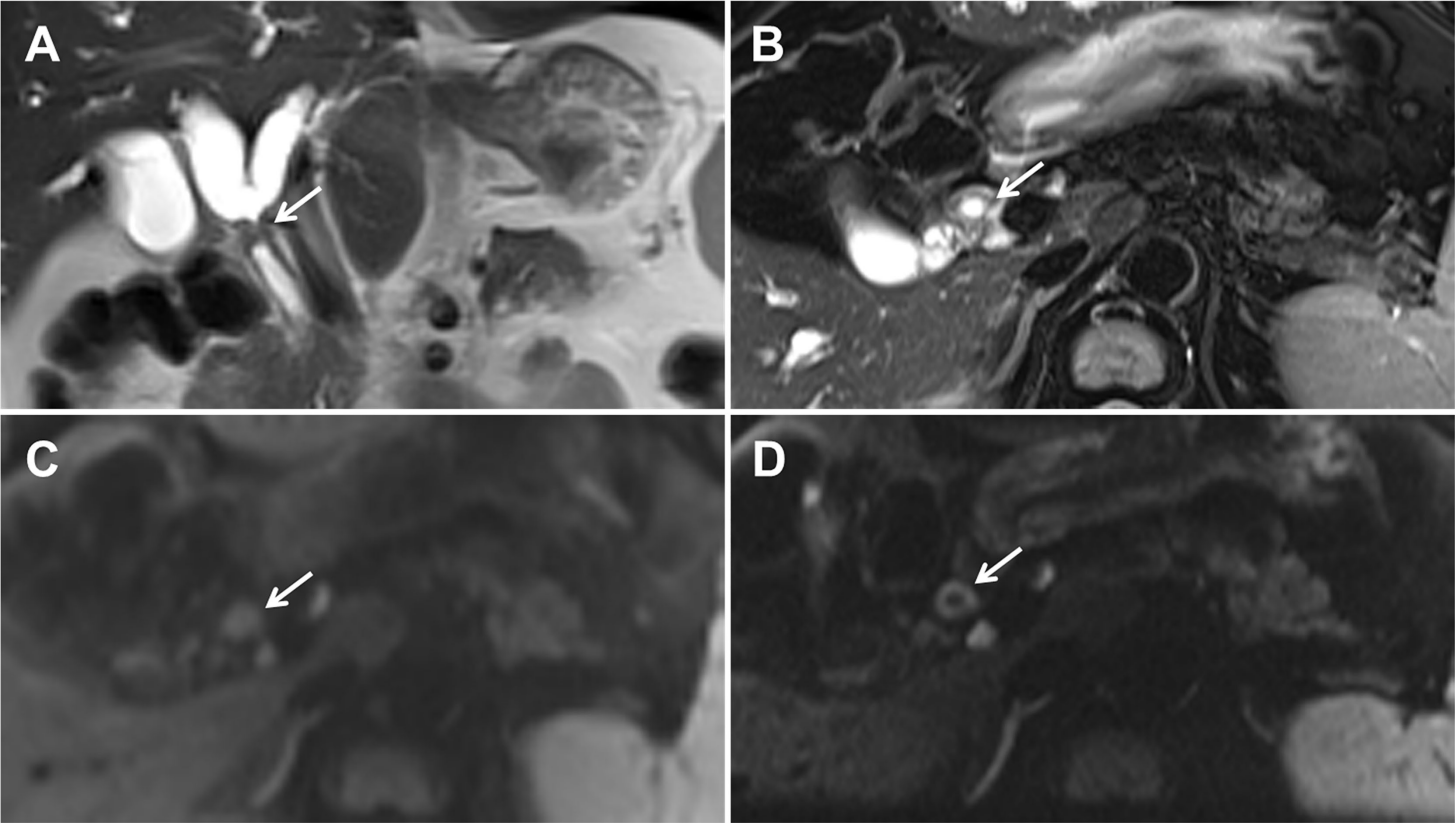
A 52-year-old man with Bismuth-Corlette type I hilar cholangiocarcinoma. Coronal **(A)** and axial **(B)** T2-weighted magnetic resonance imaging images demonstrating the hepatic duct with wall thickening and mild hyperintensities. Conventional echo-planar (c-EPI) diffusion-weighted imaging (DWI) **(C)** and zoomed echo-planar imaging (z-EPI) DWI **(D)** was obtained at b=800 s/mm^2^. The common hepatic duct lesion showed hyperintensities. In addition, the thickened common hepatic duct wall is better delineated on the z-EPI DWI image (arrow in **D**) compared with the c-EPI DWI image (arrow in **C**).

z-EPI DWI indicated significantly higher image quality scores compared with c-EPI DWI (**Table 2**). The hepatic hilar region images from z-EPI DWI exhibited better anatomic structural delineations (2.94 ± 0.25), lesion conspicuities (2.91 ± 0.27) and overall image qualities (8.47 ± 0.81) compared with the images from c-EPI DWI (anatomic structure visualization, 2.41 ± 0.49; lesion conspicuity, 2.28 ± 0.63; overall image quality, 7.28 ± 1.17) (all p<0.05). A better delineation of lesion bile ductular and luminal walls was noted in 4 patients using z-EPI DWI (**Figure 1**). However, there was no statistically significant differences for artifacts from the two image data sets (p=0.876). The overall inter-observer agreement between the two readers was fair to substantial and the weighted κ values between the readers ranged from 0.500 to 0.644 for z-EPI DWI and from 0.319 to 0.768 for c-EPI DWI (**Table 3**).

**Table 2:** Image quality scores for the comparison of c-EPI DWI and z-EPI DWI.

	Anatomic structure visualization	Lesion conspicuity	Artifacts	Overall image quality
z-EPI DWI (b=800 s/mm^2^)				
Reader1	2.94 ± 0.25	2.94 ± 0.25	2.75 ± 0.58	8.63 ± 0.72
Reader2	2.94 ± 0.25	2.88 ± 0.34	2.69 ± 0.60	8.50 ± 0.89
Average	2.94 ± 0.25	2.91 ± 0.27	2.72 ± 0.55	8.47 ± 0.81
**c-EPI DWI (b=800 s/mm^2^)**				
Reader1	2.44 ± 0.51	2.31 ± 0.60	2.69 ± 0.60	7.44 ± 1.03
Reader2	2.38 ± 0.50	2.19 ± 0.75	2.68 ± 0.60	7.19 ± 1.33
Average	2.41 ± 0.49	2.28 ± 0.63	2.68 ± 0.57	7.28 ± 1.17
*P* value*	0.001	0.001	0.876	0.002

c-EPI, conventional echo-planar imaging; z-EPI, zoomed echo-planar imaging; DWI, diffusion-weighted imaging.

Data are mean ± standard deviation.

*Wilcoxon signed-rank test was performed between c-EPI DWI and z-EPI DWI sequences using averaged image quality scores of two readers.

**Table 3:** Inter-reader agreements of qualitative image quality scores.

	z-EPI DWI (b=800 s/mm^2^)	c-EPI DWI (b=800 s/mm^2^)
Anatomic structure visualization	0.636 (0.000,1.000)	0.647 (0.314,1.000)
Lesion conspicuity	0.636 (0.000,1.000)	0.595 (0.284,0.893)
Artifacts	0.644 (0.213,1.000)	0.768 (0.407,1.000)
Overall image quality	0.500 (0.106,0.812)	0.319 (0.000,0.617)

Data in parentheses represent 95% confidence intervals.

### Quantitative Image Analysis

Hilar cholangiocarcinoma lesion sizes on T_2_WI were 18.26 ± 9.55 mm. The lesion sizes were 15.97 ± 9.42 mm on z-EPI DWI and 14.04 ± 9.22 mm on c-EPI DWI, respectively. However, there were no statistically significant differences among the two DW image data sets and T_2_WI (p=0.499). Further, following the calculations, the ratio of differences regarding hilar cholangiocarcinoma lesion sizes on T_2_WI and DWI were significantly lower (p=0.018) on z-EPI DWI (0.14 ± 0.77) compared with those noted on c-EPI DWI (0.26 ± 0.16) (**Table 4**).

**Table 4:** The ratio of differences regarding hilar cholangiocarcinoma lesion sizes on T_2_WI and DWI (b=800 s/mm^2^).

	z-EPI DWI	c-EPI DWI	*P*
The ratio of differences in sizes (Size_T2WI_-Size_DWI_)/Size_T2WI_	0.14 ± 0.77	0.26 ± 0.16	0.018

c-EPI, conventional echo-planar imaging; z-EPI, zoomed echo-planar imaging; DWI, diffusion-weighted imaging; T_2_WI, T2-weighted imaging.

There were no significant differences between c-EPI DWI and z-EPI DWI in the ADC values of hilar cholangiocarcinoma lesions ((1.19 ± 0.14)×10^-3^ mm^2^/s vs. (1.17 ± 0.15)×10^-3^ mm^2^/s. p=0.48) (n = 8).

### Image Analysis

**Figure 2:**
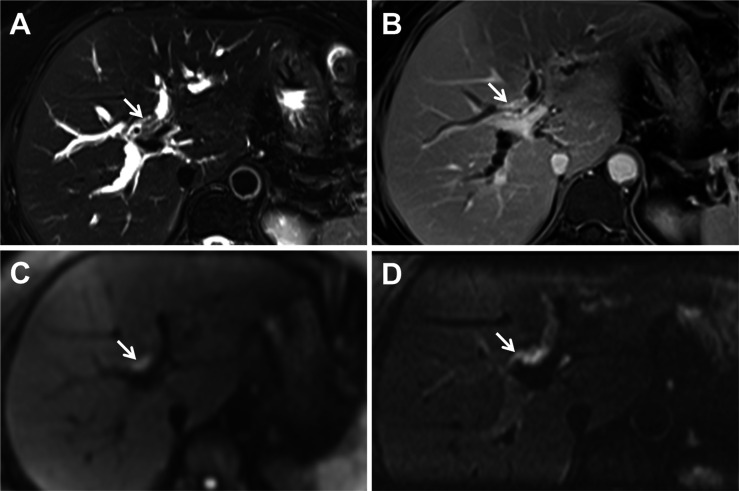
A 54-year-old woman with Bismuth-Corlette type IIIa hilar cholangiocarcinoma. Axial **(A)** T2-weighted magnetic resonance imaging images indicating the hepatic duct with wall thickening and mild hyperintensities (arrow). **(B)** Gadoxetic acid-enhanced delay phase image shows thickening of the bile duct at the hepatichilum and the right hepatic ducts (arrow). Conventional echo-planar (c-EPI) diffusion-weighted imaging (DWI) **(C)** and zoomed echo-planar imaging (z-EPI) DWI **(D)** were obtained at b=800 s/mm^2^. The lesion bile duct at the hepatic hilum indicated hyperintensities. Both readers interpreted this tumor as type II on the basis of c-EPI DWI **(C)**. However, based on z-EPI DWI, the tumor was clearly observed at the hepatic hilum and the right hepatic ducts were noted as areas of hyperintensity. Both readers interpreted this tumor as type IIIa on z-EPI DWI.

All 16 tumors were slightly hyperintense compared with liver parenchyma at b=800 sec/mm^2^ DW imaging. The use of z-EPI DWI resulted in accurate diagnosis of the Bismuth-Corlette classification of 15 tumors (15/16, 93.75%) for both of the two observers, whereas the use of c-EPI DWI resulted in correct diagnosis of 12 tumors (12/16, 75.00%) (**Table 5**). Among tumors that were misclassified, three were misclassified with c-EPI DWI but were correctly classified with z-EPI DWI by both observers (classification changed from type II to IIIa (**Figure 2**), n=2; from type IIIa to IV, n=1).

**Table 5:** Correlation of Bismuth Corlette Classification of Hilar Cholangiocarcinoma with the Pathological Findings using the DWI Study Interpretation.

Observer and Bismuth-Corlette Classification	Pathological Tumor Classification					
	Ⅰ	Ⅱ	IIIa	IIIb	IV	Accuracy (%)
Reader 1						
I	4/4	0	0	0	0	75.00/93.75
II	0	2/2	2/0	1/1	0	
IIIa	0	0	1/3	0	1/0	
IIIb	0	0	0	3/3	0	
IV	0	0	0	0	2/3	
Reader 2						
I	4/4	0	0	0	0	75.00/93.75
II	0	2/2	2/0	1/1	0	
IIIa	0	0	1/3	0	1/0	
IIIb	0	0	0	3/3	0	
IV	0	0	0	0	2/3	
Total	4	2	3	4	3	

Data are numbers of tumors for c-EPI DWI/for z-EPI DWI.

## Discussion

For abdominal MRI, DWI has been applied to detect and characterize various abdominal malignant lesions because it can offer high contrast between tumor and surrounding normal tissue. In the preoperative evaluation of hilar cholangiocarcinoma, the additional application value of DWI in the detection of tumor extent along the bile duct and liver invasion has been investigated (3). It is of paramount importance to obtain a precise preoperative assessment of tumor extension to plan to achieve microscopically complete resection of hilar cholangiocarcinoma. However, abdominal DWI using ss-EPI is often disturbed by the adjacent air in the stomach and intestines and abdominal organ and aorta motion during scanning. In addition, abdominal DWI is known to be technically challenging regarding distortion artifacts in the phase-encoding direction. The z-EPI DWI method has potentially better advantages over conventional single-shot EPI for using two-dimensional spatial-selective radiofrequency pulses to obtain reduced volumes. This technique can achieve higher resolution and reduce distortions without introducing unfolding artifacts (9, 18). To the best of our knowledge, this is the first MRI study to evaluate the use and clinical application of z-EPI DWI to study the hepatic hilar region.

In the present study, the comparison of the image quality of z-EPI DWI and c-EPI DWI was performed in order to evaluate hilar cholangiocarcinoma lesions. The results indicated that the hepatic hilar region was better delineated with the z-EPI DWI using anatomic and structural visualization, lesion conspicuity and overall image quality (all p<0.05) compared with the c-EPI DWI. For 4 patients among them, the thickened lesion bile duct walls and the lumens could be distinctly delineated on z-EPI DWI while not be identified on c-EPI DWI. Our study results showed that z-EPI DWI indicated significantly higher scores for hilar cholangiocarcinoma lesion conspicuity than c-EPI DWI, and consistent with previous abdominal organ research results (19). However, in our study the difference for image artifacts was not significant between z-EPI DWI and c-EPI DWI, which may be that hepatic hilar region with high position is less influenced by adjacent air and motion from the stomach and intestines. In a word, z-EPI DWI can be used to get more image information for suspicious hilar cholangiocarcinoma cases in routine clinical practice.

Quantitative image analysis showed that there were no statistically significant differences of the lesion sizes from the two DW image data sets and T_2_WI. Further, the results showed that the ratio regarding the differences in the hilar cholangiocarcinoma lesion sizes on T_2_WI and DWI was significantly lower on z-EPI DWI compared with that of c-EPI DWI (*P*=0.018), indicating that lesion sizes on z-EPI DWI were closer to those noted on T_2_WI than those on c-EPI DWI. These results further indicate a better visualization of anatomic structures and lesion conspicuities with the z-EPI DWI.

Accurate diagnosis of the Bismuth-Corlette classification of hilar cholangiocarcinoma is helpful for treatment selection. The results of the present study indicated that the use of z-EPI DWI resulted in high accurate diagnosis of the Bismuth-Corlette classification, and the Bismuth-Corlette classification were correctly classified with z-EPI DWI owing to it could provide more imaging information. z-EPI DWI may be an effective supplement for detecting the extent of bile duct lesions.

The present study contains certain limitations. Initially, a limited spectrum of diseases was included. A substantial type of diseases will be included in future studies for subsequent investigations. Furthermore, only two b values (50 and 800 s/mm^2^) were used in the present study to reduce imaging times in the clinical setting. In addition, we did not perform a quantitative analysis of the ADC values of all lesions as it was difficult to accurately measure because of their relatively small sizes, especially for hilar cholangiocarcinoma with the presentation of the thickened wall of the bile duct. Fourth, the technique itself has the limitation of not capturing pathologies outside the zoomed FOV.

## Conclusion

In conclusion, z-EPI DWI indicated remarkable improvements in overall image quality for the assessment of hilar cholangiocarcinoma lesions compared with the c-EPI DWI. In addition, z-EPI DWI provided better anatomic structure delineations and lesion conspicuities and was superior in detecting and delineating lesions compared with the c-EPI method. Therefore, z-EPI DWI should be performed complementary to c-EPI DWI in clinical settings. These findings may aid radiologists to evaluate hilar cholangiocarcinoma lesions in greater detail.

## Data Availability Statement

The raw data supporting the conclusions of this article will be made available by the authors, without undue reservation.

## Ethics Statement

The studies involving human participants were reviewed and approved by the Institutional Review Board of the First Affiliated Hospital of Zhengzhou University. The patients/participants provided their written informed consent to participate in this study.

## Author Contributions

Guarantor of integrity of the entire study: JL and JC; study concepts and design: JL and JZ; literature research: JL and JZ; data collection: MH, YR, MX, and YL; data analysis: JL and MH; manuscript preparation: JL; manuscript review: JL, JC, and JZ. All authors read and approved the final version of the manuscript.

## Conflict of Interest

JZ is employed by MR Collaboration, Siemens Healthcare Ltd., Beijing, China.

The remaining authors declare that the research was conducted in the absence of any commercial or financial relationships that could be construed as a potential conflict of interest.

## Publisher’s Note

All claims expressed in this article are solely those of the authors and do not necessarily represent those of their affiliated organizations, or those of the publisher, the editors and the reviewers. Any product that may be evaluated in this article, or claim that may be made by its manufacturer, is not guaranteed or endorsed by the publisher.

## Abbreviations

z-EPI, zoomed echo-planar imaging; c-EPI, conventional echo-planar imaging; DWI, diffusion-weighted imaging; T2WI, T2-weighted imaging; T1WI,T1-weighted imaging; DCE, dynamic contrast-enhanced.
