# Corrigendum: The evaluation of zoomed echo-planar diffusion-weighted magnetic resonance imaging with two-dimensional spatial-selective radiofrequency excitation pulses in patients with hilar cholangiocarcinoma

**DOI:** 10.3389/fonc.2022.989870

**Published:** 2022-09-21

**Authors:** Jingjing Liu, Mengyue Huang, Yanan Ren, Man Xu, Yinhua Li, Jingliang Cheng, Jinxia Zhu

**Affiliations:** ^1^ Department of Magnetic Resonance Imaging, The First Affiliated Hospital of Zhengzhou University, Zhengzhou, China; ^2^ MR Collaboration, Siemens Healthcare Ltd., Beijing, China

**Keywords:** hilar cholangiocarcinoma, zoomed EPI, diffusion-weighted imaging, magnetic resonance imaging, image quality

In the published article, there was an error in **Table 1** as published. The acquisition time of z-EPI DWI in **Table 1** was displayed as “3min45s”. The correct statement is “3min18s~6min11s (depending on breathing pattern)’’. The acquisition time of axial T_2_WI in **Table 1** was displayed as “2min50s”. The correct statement is “3min44s~4min28s (depending on breathing pattern)”. The corrected **Table 1** and its caption “Magnetic resonance imaging parameters” appear below.

**Table 1:** Magnetic resonance imaging parameters.

Imaging parameters	c-EPI DWI	z-EPI DWI	Coronal T_2_WI	Axial T_2_WI	T_1_WI/DCE
TR [ms]	4500	2000	1400	3100	3.9
TE [ms]	56	61	67	87	1.89
FOV [mm^2^]	350 × 292	230 × 120	360 × 360	380 × 380	380 × 309
Reconstructed voxel size (mm^3^)	2.2 × 2.2 × 5	1.5 × 1.5 × 5	1.4 × 1.4 × 5	1.2 × 1.2×5	0.7 ×0.7 ×3
Acquisition matrix	158 × 121	154 × 50	256 ×256	320 × 320	288× 187
Slice thickness [mm]	5	5	5	5	3
b-values [s/mm^2^]	50/800	50/800	/	/	/
Parallel acceleration factor	2	2	3	2	2
Bandwidth (Hz/px)	1978	1248	698	710	1090
Respiration control	Free-breathing	Trigger	Breath-hold	Trigger	Breath-hold
Acquisition time	1min52s	3min18s~6min11s (depending on breathing pattern)	28s	3min44s~4min28s (depending on breathing pattern)	17s

The authors apologize for this error and state that this does not change the scientific conclusions of the article in any way. The original article has been updated.

## Publisher’s note

All claims expressed in this article are solely those of the authors and do not necessarily represent those of their affiliated organizations, or those of the publisher, the editors and the reviewers. Any product that may be evaluated in this article, or claim that may be made by its manufacturer, is not guaranteed or endorsed by the publisher.

